# Information Matching: How Regulatory Focus Affects Information Preference and Information Choice

**DOI:** 10.3389/fpsyg.2021.618537

**Published:** 2021-05-28

**Authors:** Xiaomei Wang, Jia Wang

**Affiliations:** School of Media Studies and Humanities, Zhejiang University City College, Hangzhou, China

**Keywords:** regulatory focus, explicit preference, implicit preference, information choice, intermediary role

## Abstract

Individuals often prefer information that matches their needs. In this study, we aimed to explore the relationship between regulatory focus and information preference. Specifically, we investigated the effects of promotion-focused information and prevention-focused information on explicit and implicit information preferences and choice behavior, and examined the mediating roles of information preference. In Experiment 1, we found that prevention-focused individuals were more likely to choose functional information, whereas promotion-focused people were more likely to choose hedonic information. However, there was no significant relationship between regulatory focus and explicit preference and no mediating effect of explicit information preference. In Experiment 2, we found that promotion-focused individuals had a greater implicit preference for hedonic information than did prevention-focused individuals. Implicit information preference mediated the influence of regulatory focus on information choice. The findings of this study may help us understand the psychological mechanism underlying information preference and have important implications for information dissemination.

## Introduction

In the field of *consumer behavior* studies, products are generally divided into *hedonic* and *functional* ones. While consuming hedonic products, people usually focus on these products' *experiential aspects*, mainly because they are manufactured to generate feelings of happiness, excitement, and stimulation. On the other hand, while consuming functional products, people focus on their *practical aspects*, mainly because these products are expected to be rationally consumed and are necessary to perform specific tasks (Chaudhuri, 2002). Hence, some researchers believe that information can be divided into two types, as well: *functional* and *hedonic information* (Van der Heijden, 2004). Functional information refers to instrumental and practical types of information, whereas hedonic information encompasses aesthetic and experiential types of information (Chitturi et al., 2007).

Nevertheless, due to their limited processing capacity, human beings cannot process all the information they have access to; they choose the information that they want to be processed. Then, how do motivational orientations influence information choice? Although numerous studies have documented regulatory focus and information processing of individuals (Roy and Ng, 2012; Burtscher and Meyer, 2014; Roy and Phau, 2014), there is limited research into the various effects of regulatory focus on information behavior of individuals at the explicit and implicit levels. Hence, the present study aimed to bridge this knowledge gap and to test a model that accounts for the influence of regulatory focus on information preference. In this regard, a few questions need to be addressed: Do people prefer to access information that matches their regulatory goals? Further, what roles do explicit and implicit information preferences play in the process of information choice?

## Theoretical Background

### Perspectives on Regulatory Focus and Information Preference

Higgins (1997) proposed the *Regulatory Focus Theory*: people have different tendencies regarding their goal regulation focuses, that is, *promotion* and *prevention* focuses. Promotion-focused individuals tend to hold a gain/non-gain mindset and pursue goals related to aspiration and accomplishment. By contrast, prevention-focused individuals tend to hold a non-loss/loss mindset and pursue goals related to safety and protection. Therefore, promotion-focused individuals are more sensitive to the presence of positive outcomes and are inclined to adopt approach-oriented strategies. Meanwhile, prevention-focused individuals are more sensitive to negative outcomes and are inclined to adopt avoidance-oriented strategies. As a result, the regulatory focus may influence the type of information sought (Higgins, 1999). And it is important to note that studies on regulatory focus use either a chronic trait or a motivational state to create it. Chronic focus, as a personality trait, is typically captured via standardized measures (Hong and Lee, 2008). Conversely, motivational focus, as a momentary state, can be temporarily activated via self-generated prime (asking a participant to list the duties and obligations (prevention) or the hopes and aspirations (promotion), Freitas and Higgins, 2002) or situation-generated priming (asking a participant to complete a maze task, Friedman and Förster, 2001). Current researchers utilize a dual-task paradigm (self-generated and situation-generated) to prime regulatory focus.

People with different types of regulatory focus assign different values to different types of information. Specifically, promotion-focused individuals expect to achieve goals through the hedonic dimension (i.e., aesthetic, experiential, and pleasure), whereas prevention-focused individuals expect to achieve goals through the functional dimension (i.e., utilitarian, practical, and instrumental) (Chernev, 2004; Chitturi et al., 2007). Promotion-focused goals lead people to make more emotion-driven decisions, and they prefer hedonic outcomes; prevention-focused goals lead people to make more rationality-driven decisions and prefer functional products (Pham and Avnet, 2004; Das et al., 2018). Similarly, some studies have shown that when accessing a website that contains a lot of hedonic (functional) information, promotion-focused (prevention-focused) individuals rate it more positively (Ashraf et al., 2016a,b). Hence, the regulatory focuses act as filters (Wang and Lee, 2006) individuals tend to prefer stimuli that match their regulatory focuses. In the current study, we investigated the effects of regulatory focus on information preference and choice behavior. Accordingly, we proposed the following hypotheses:

H_1a_: Promotion-focused individuals prefer hedonic information in terms of attitude and choose hedonic information in terms of behavior.

H_1b:_ Prevention-focused individuals prefer functional information in terms of attitude and choose functional information in terms of behavior.

### Capturing Implicit and Explicit Information Preferences

**Figure 1:**
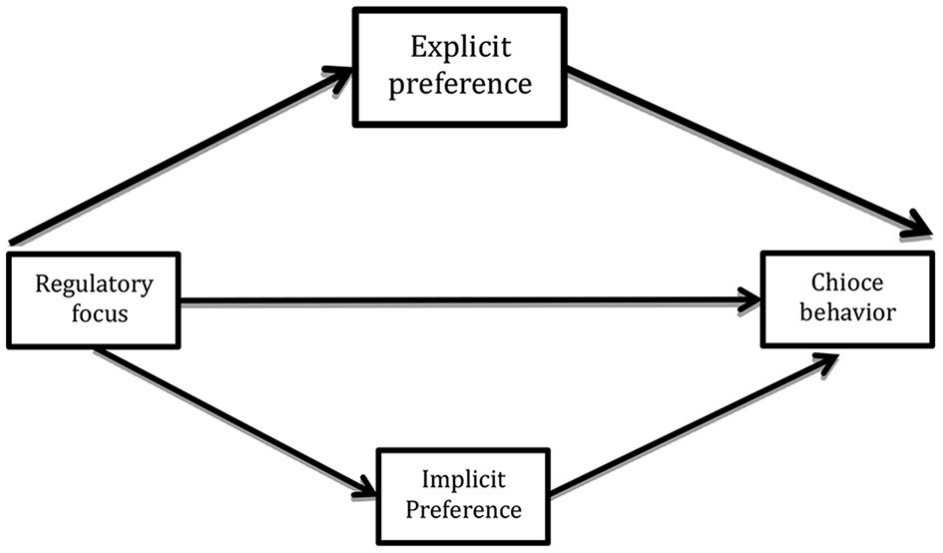
Mediation model as proposed in Hypothesis 2.

Although many established models account for the effects of regulatory focus, most of them are based on explicit measures, which are directly assessed using questionnaires or interviews and are characterized by process awareness. However, studying explicit preferences alone may not capture the complete true effect of regulatory focus. To understand the latent information preference, implicit measurement approaches, which are assessed indirectly and unconsciously, need to be adopted (Gawronski and Bodenhausen, 2006). That is, there is often a discrepancy between explicit and implicit attitudes (Steffens, 2004). Hence, information preferences must be captured via explicit and implicit attitude measures. Additionally, a study found that explicit and implicit attitudes have different predictive powers for behavior (Wilson et al., 2000). Therefore, we aimed to focus on not only explicit information preferences but also implicit information preferences. Explicit or implicit preferences are modeled as potential mediators in the relationship between regulatory focus and information choice behavior (Figure 1 illustrates the hypothesized mediation model). Our second hypothesis was as follows.

H_2_: Explicit or implicit information preference mediates the influence of regulatory focus on information choice behavior.

## Experiment 1

### Participants and Design

In this experiment, 72 (38.9% males and 61.1% females) university students participated in exchange for course credit. They were randomly assigned to promotion-focused and prevention-focused groups. On completing the experiment, all the participants received a gift worth CNY 15 yuan.

### Procedure and Materials

The experiment consisted of three phases. The first phase comprised a dual-task paradigm to induce regulatory focus. Specifically, the first task was a recall task. Participants in the promotion-focused group were asked to write down their ideals and aspirations, and those in the prevention-focused group were requested to write down their oughts and obligations (Freitas and Higgins, 2002; Wang et al., 2020). After this, all participants were asked to complete a paper–pencil maze task to further understand their promotion or prevention focuses (Friedman and Förster, 2001).

In the second phase, participants completed a three-item questionnaire, which assesses the effects of priming manipulations of regulatory focus. These questions were designed to include two opposite options on a 7-point Likert scale, with options ranging from 1 (emphasis on ideals) to 7 (emphasis on oughts), such as “I'm more willing to do what I want (ideals)” vs. “I'm more willing to do what everyone agrees to be right (oughts)” (Pham and Avnet, 2004).

In the third phase, participants first completed a questionnaire on information choice behavior. The questionnaire consisted of four different choices of scenarios, and each scenario provided two possible options. The scenarios began with the statement: “…here are four different options. If you had 10 min, which one would you choose to watch? Please choose between options A and B based on your current thoughts: (A) economic information; (B) entertainment information.” Participants chose one of these two options to represent their information choice preferences (Wang et al., 2020). After that, participants completed a questionnaire that assessed explicit information preference. The questionnaire was adapted from the Attitude Scale used in Blankenship and Wegener's (2008) study. Participants indicated their explicit preference of hedonic and functional information, respectively on five semantic 7-point Likert scales, ranging from 1 (harmful, foolish, bad, unfavorable, and undesirable) to 7 (beneficial, wise, good, favorable, and desirable). The difference in explicit preference for hedonic and functional information was used as a dependent variable, and this variable was calculated by subtracting explicit preference for functional information from the hedonic information. The Cronbach's α coefficient was 0.86.

### Results

#### Manipulation Checks

The results of the independent sample *T*-test showed that in the prevention focus condition, participants tended to place a higher value on oughts compared to ideals, *M*_*promotion*_ = 2.58 (*SD* = 0.63); *M*_*prevention*_ = 4.36 (*SD* = 0.84); *t*(70) = −10.16; *p* < 0.001, *Cohen's d* = 2.40 (Cohen, 1988). We thus deemed that the regulatory focus manipulation was successful.

#### Information Choice Behavior and Explicit Preference

According to the results of a *t*-test, there was a statistically significant difference in information choice behavior between both groups: *M*_*promotion*_ = 1.17 (*SD* = 0.21); *M*_*prevention*_ = 1.36 (*SD* = 0.30); *t*(70) = −3.13; *p* < 0.003, *Cohen's d* = 0.73. Specifically, compared to promotion-focused individuals, prevention-focused individuals showed a higher choice preference for functional information than for hedonic information. However, there was no significant effect of regulatory focus on explicit preference for both groups: *M*_*promotion*_ = −0.88 (*SD* = 1.19); *M*_*prevention*_ = −1.26 (*SD* = 1.22); *t*(70) = 1.35; *p* > 0.05, *Cohen's d* = 0.32. In summary, regulatory focus affected information choice behavior but not explicit information preference, thus partially supporting our first hypothesis.

#### Mediating Effect of Explicit Information Preference

According to the second hypothesis, explicit information preference mediates the influence of regulatory focus on information choice. To test this, we constructed a mediation path model (Preacher and Hayes, 2008). Information choice was regressed on regulatory focus and information explicit preference (see Table 1). The bootstrap version of the Sobel test showed that the full regression model accounted for more than 69% of the variance in information choice, *p* < 0.05; however, there was an insignificant, indirect effect of regulatory focus on information choice through explicit information preference, *ab* = 0.07, bias corrected 90% CI (−0.02, 0.18), 5,000 resamples.

**Table 1:** Results of the regression analysis predicting information choice.

**Predict**	**Δ*R^**2**^***	**β**	**90% CI B**
Step 1	0.12^*^		
Regulatory focus		0.70	(0.33, 1,07)
Step 2	0.15^*^		
Regulatory focus		0.64	(0.27, 1.01)
Explicit preference		−0.18	(-0.37, 0.08)

* *p < 0.05*.

## Experiment 2

### Participants and Design

In Experiment 2, 68 (29 male and 39 female) university students participated in exchange for course credit. They were randomly assigned to promotion-focused and prevention-focused groups. On completing the experiment, all the participants received a gift worth RMB 15 yuan.

### Procedure and Materials

The procedure adopted in Experiment 2 was essentially the same as that adopted in Experiment 1, with the exception of the dependent variable implicit information preference instead of explicit information preference. The Implicit Association Test (IAT) was conducted to measure implicit preferences for hedonic information and functional information. The test contains 7 blocks with 20 or 40 trials (stimuli associated with positive or negative attributes and stimuli associated with hedonic or functional information) (Wang et al., 2020). For example, in one of the blocks, participants categorized positive words and hedonic information as quickly as possible by pressing the “E” key; similarly, they categorized negative words and functional information by pressing the “I” key. In another block, participants categorized positive words and functional information by pressing the “E” key, and negative words and hedonic information by pressing the “I” key. Details of the blocks are shown in the Table 2. The implicit effect was calculated using D scores (Greenwald et al., 2003). A high D value indicates that hedonic information was more preferred than functional information.

**Table 2:** Sequence of blocks for the IAT experiment.

**Blocks**	**Categories for “E” key**	**Categories for “I” key**	**Number of trials**
1. Single categorization of target word (practice)	Hedonic information	Functional information	20
2. Single categorization of target word (practice)	Positive, e.g., happy	Negative, e.g., hypocrisy	20
3. Combined categorization (practice)	Positive/hedonic information	Negative/functional information	20
4. Combined categorization (test)	Positive/hedonic information	Negative/functional information	40
5. Single categorization of target word (reversed)	Functional information	Hedonistic information	20
6. Combined categorization (practice, reversed)	Positive/functional information	Negative/hedonic information	20
7. Combined categorization (test, reversed)	Positive/function information	Negative/hedonic information	40

The D score calculation program contained seven stages, and the procedure automatically recorded the correct rate and latency for responses. The IAT scoring procedures were as follows (Greenwald et al., 2003):

(1) Delete trials >10,000 ms;
(2) Delete participants for whom more than 10% of trials have latency <300 ms;
(3) Calculate mean latency of correct responses for each combined Stage (3, 4, 6, 7);
(4) Replace each error latency with “Stage mean + 600 ms”;
(5) Calculate the mean latency for each of Stages 3, 4, 6, and 7;
(6) Compute the two mean differences (Meanstage 6 – Meanstage 3) and (Meanstage 7 – Meanstage 4);
(7) Divide each difference score by its associated standard deviation; *D* = the equal-weight average of the two resulting ratios.

### Results

#### Descriptive Statistics for Response Times

A paired sample *t*-test of IAT response times (*M*_*compatibility*_ = 1,314.96, *SD* = 385.59; *M*_*incompatibility*_ = 1,409.63, *SD* = 365.04; *t*(66) = −2.10; *p* < 0.05, *Cohen's d* = 0.25) showed that participants' response times for compatible tasks (hedonic information and positive words, functional information and negative words) were significantly shorter those that for incompatible tasks (functional information and positive words, hedonic information and negative words).

#### Implicit Information Preference

A *t*-test showed that regulatory focus had a significant effect on information implicit preference (*M*_*promotion*_ = 0.23, *SD* = 0.42; *M*_*prevention*_ = 0.03, *SD* = 0.40; *t*(66) = 2.02, *p* < 0.05, *Cohen's d* = 0.49). A larger D value indicated a stronger association between hedonic information and the positive attribute, whereas a smaller D value indicated a stronger association between functional information and the positive attribute. In other words, promotion-focused individuals had a greater preference for hedonic information than did prevention-focused individuals.

#### Mediating Effect of Implicit Information Preference

According to the second hypothesis, implicit information preference mediates the influence of regulatory focus on information choice. To test this, we constructed a mediation path model like in Experiment 1. Information choice was regressed on regulatory focus and information implicit preference (see Table 3). Using the bootstrap version of the Sobel test, we found the full regression model accounted for more than 65% of the variance in information choice, *p* < 0.01; and there was a significant, indirect effect of regulatory focus on information choice through implicit information preference, *ab* = 0.11, bias corrected 90% CI (0.003, 0.230), 5,000 resamples. The findings suggest that individuals in the promotion-focused group were more inclined to choose hedonic information (rather than functional information) than were individuals in the prevention-focused group, because they had a greater implicit preference for hedonic information.

**Table 3:** Results of the regression analysis predicting information choice.

**Predict**	**Δ*R*^**2**^**	**β**	**90% CI B**
Step 1	0.11^**^		
Regulatory focus		0.65	(0.25, 1.06)
Step 2	0.15^**^		
Regulatory focus		0.54	(0.12, 0.95)
Implicit preference		−0.22	(−0.43, −0.01)

** *p < 0.01*.

## Discussion

In this study, we explored whether regulatory focus influences information preference (information explicit/implicit preference) and information choice behaviors. Our results showed that individuals' regulatory focus affected their information choice and information preference (more specifically, implicit preferences rather than explicit preferences). Promotion-focused individuals preferred hedonic information, whereas prevention-focused individuals preferred functional information. The finding on the effect of regulatory focus on information preference is consistent with the findings of previous studies. Chernev (2004) revealed that people pay more attention to items that match their regulatory focus. Roy and Ng (2012) also pointed out the association between promotion-focused (vs. prevention-focused) goals and hedonic (vs. functionality) goals. Compared to prevention-focused individuals, promotion-focused individuals were tended to seek hedonic information. There is an asymmetry between the two types of regulatory focuses, whereby promotion-focused individuals are more likely to be activated by “ideals” and prevention-focused ones by “oughts” (Idson et al., 2004). The desire induced by the promotion makes people seek risks, prefer hedonic products, and easily adopt heuristic strategies in decision-making. Contrastingly, focusing on negative outcomes leads prevention-focused people to believe that something is wrong and that an action needs to be taken to rectify the situation. As a result, individuals evaluate the situation carefully taking care to avoid unintended consequences, prefer functional products, and require substantive information in their decision-making (Chernev, 2004).

Our results also showed that regulatory focus affected information choice behaviors through implicit information preference, but not explicit information preference. Most studies suggest that the prediction of people's behavioral intention relies on explicit measurement. However, explicit preferences refer to an individual's judgment, which can be consciously assessed by self-reported methods such as surveys. Owing to certain social norms or expectations, most people often cannot or do not want to explicitly express their true preferences (Greenwald et al., 2009). Implicit preference refers to an individual's judgment, which based on an accurate, automated assessment without any intention. Implicit preferences can be assessed through implicit tests such as the IAT, which is a widely-used measure (Axt et al., 2014). One of the main reasons for its widespread use is that researchers believe it can predict relevant outcomes beyond explicit measures (Buttrick et al., 2020; Irving and Smith, 2020). Some studies also suggest that when individuals are unwilling or unable to express themselves, implicit preference may be a better predictor of behavior compared to explicit preference (Sekaquaptewa et al., 2003). This is not to understate the importance of explicit measurements; in fact, self-report measures are a vital predictor of behaviors (Kormos and Gifford, 2014). Nevertheless, it is possible that implicit measures can incrementally predict behaviors that are more controllable or unspontaneous. Previous studies have shown that negative implicit evaluation is related to more negative, non-deliberate social behavior, such as aggressive behaviors in social interactions with peers (Lansu, 2018), and nonverbal communication (Dovidio et al., 2002). In addition, a recent study indicated that compared to explicit attitudes, implicit transgender attitudes predict beliefs and experiences, including gender essentialism, contact with transgender people, and support for transgender-related policies (Axt et al., 2020). Our findings partially confirmed the hypothesis that implicit information preference mediates the influence of regulatory focus on (not explicit) information choice behavior. Individuals in the promotion-focused group condition were inclined to select hedonic information rather than functional information than were individuals in the prevention-focused group, because they had a greater implicit preference for hedonic information.

## Implications

The Regulatory Focus Theory received widespread attention following its introduction. Nevertheless, its application in the field of information preference remains limited. The current study examined the impact of individuals' regulatory focus type on their information choice behavior and information preferences, in particular, the mediating role of implicit preference. These findings expand the scope of research into the Regulatory Focus Theory and help us understand the underlying mechanisms of individuals' attitudes and behavior regarding information.

Further, the findings of this study may help businesses and marketing professionals to device effective marketing strategies to improve the audience's acceptance of specific types of information. For example, if merchants require target consumers to accept a product's functional information, they may stimulate the consumers' prevention focus; however, to ensure acceptance of hedonic information, merchants should pursue efforts to stimulate their consumers' promotion focus. Hence, consumer acceptance can be effectively increased by adopting different marketing strategies for different types of information.

## Limitations And Future Directions

This study has several shortcomings. First, the participants were all students who lacked work experience. Such individuals are more susceptible to priming effects than are social workers. It remains to be answered whether the effect of regulatory focus on information preference would be equally valid in other settings. Future studies are needed to further verify its external validity. Second, a cross-sectional study was conducted to examine the influence of situational regulatory focus on information preference. However, regulatory focus could be a situational or trait state; therefore, pre-existing trait regulatory focus may have affected the reliability of the results. Moreover, considering the difficulty in testing the mediation mechanism, further longitudinal studies are needed to improve the robustness of studies. Finally, our study only addressed information choice behavior, not the actual behavior. In consideration of the possible difference between self-reported information choice behavior and actual behavior, the relationship between regulatory focus and actual information behavior should be tested in future studies.

## Data Availability Statement

The original contributions presented in the study are included in the article/supplementary material, further inquiries can be directed to the corresponding authors.

## Ethics Statement

Ethical review and approval was not required for the study on human participants in accordance with the local legislation and institutional requirements. The patients/participants provided their written informed consent to participate in this study.

## Author Contributions

XW formulated the research goals and aims, developed and designed the methodology, collected the data, and acquired the financial support for the project leading to this publication. JW prepared the published work and made statistical analyses, conducted data collection and edited. All authors were involved in the writing of the theoretical background and discussion sections.

## Conflict of Interest

The authors declare that the research was conducted in the absence of any commercial or financial relationships that could be construed as a potential conflict of interest.
